# Assessment of the accreditation standards of the Central Board for Accreditation of Healthcare Institutions in Saudi Arabia against the principles of the International Society for Quality in Health Care (ISQua)

**DOI:** 10.4103/0256-4947.67082

**Published:** 2010

**Authors:** Abdullah AlKhenizan, Charles Shaw

**Affiliations:** a From the Department of Family Medicine and Polyclinics, King Faisal Specialist Hospital and Research Centre, Riyadh, Saudi Arabia; b From the European Society for Quality in Healthcare, Limerick, Ireland

## Abstract

**BACKGROUND AND OBJECTIVES:**

Accreditation is usually a voluntary program, in which trained external peer reviewers evaluate health care organization’s compliance with pre-established performance standards. Interest in accreditation is growing in developing countries, but there is little published information on the challenges faced by new programs. In Saudi Arabia, the Central Board for Accreditation of Healthcare Institutions (CBAHI) was established to formulate and implement quality standards in all health sectors across the country. The objective of this study was to assess a developing accreditation program (CBAHI standards) against the International Society for Quality in Health Care (ISQua) principles to identify opportunities for improvement of the CBAHI standards.

**METHODS:**

A qualitative appraisal and assessment of CBAHI standards was conducted using the published ISQua principles for accreditation standards.

**RESULTS:**

The CBAHI standards did not describe the process of development, evaluation or revision of the standards. Several standards are repetitive and ambiguous. CBAHI standards lack measurable elements for each standard. CBAHI standards met only one criterion (11.1%) of the Quality Improvement principle, two criteria (22.2%) of Patient/Service User Focus principle, four criteria (40%) of the Organizational Planning and Performance principle, the majority (70%) of the criteria for the safety principle, only one criteria (7.1%) for the Standards Development principle, and two criteria (50%) of the Standards Measurement principle.

**CONCLUSIONS:**

CBAHI standards need significant modifications to meet ISQua principles. New and developing accreditation programs should be encouraged to publish and share their experience in order to promote learning and improvement of local accreditation programs worldwide.

Accreditation is usually a voluntary program, sponsored by a non-governmental agency (NGO), in which trained external peer reviewers evaluate a health care organization’s compliance with pre-established performance standards.^1^ Quality standards for hospitals and other medical facilities were first introduced in the United States in the ‘Minimum Standard for Hospitals ” developed by the American College of Surgeons in 1917. After World War II, increased world trade in manufactured goods led to the creation of the International Standards Organization (ISO) in 1947.^2^ Accreditation formally started in the United States with the formulation of Joint Commission on Accreditation of Healthcare Organizations (JCAHO) in 1951. This model was exported to Canada and Australia in the 1960s and 1970s and reached Europe in the 1980s. Accreditation programs interest is growing rapidly among developing countries.^3^ There are other forms of systems used worldwide to regulate, improve and market health care providers and organizations including Certification and Licensure. Certification involve formal recognition of compliance with set standards (e.g. ISO 9000 standards) validated by external evaluation by an authorized auditor. Licensure involves a process by which a government authority grants permission, usually following inspection against minimal standards, to an individual practitioner or healthcare organization to operate in an occupation or profession.^1^ Although the terms accreditation and certification are often used interchangeably, accreditation usually applies only to organizations, while certification may apply to individuals, as well as to organizations.^2^

The Central Board for Accreditation of Healthcare Institutions (CBAHI) in Saudi Arabia was formed in 2005 based on the recommendation of the Council of Health Services in Saudi Arabia. CBAHI was established to formulate and implement quality Standards in all health sectors all over the Kingdom of Saudi Arabia.^4^ ISQua, The International Society for Quality in Health Care is a not-for-profit organization that was established in 1985 to drive continuous improvement in the quality and safety of healthcare worldwide through education, research, collaboration and the dissemination of evidence-based knowledge. ISQua provides internationally recognized principles for healthcare standards.^5^ Several sets of healthcare standards used in Australia, Canada, Egypt, England and the standards of the Joint Commission International, USA, have already been successfully accredited by ISQua.^6^

In 1994, Saudi Aramco established the Saudi Medical Services Organization Standards. Private and governmental hospitals had to meet Aramco standards to be accepted as referral health care institutions for Aramco’s employees. In 2001, The Council for the development of health services in the Makkah region was established. One of the main products of this council was the establishment of the Makkah Region Quality Program (MRQP) in 2003, which involved written standards to be met by governmental and private hospitals working in the Makkah region (57 hospitals). These standards were based on JCAHO, and ARAMCO standards. In October 2005, the minister of health established the CBAHI in Saudi Arabia. CBAHI plans to start the accreditation process in the year 2010.^6^ Several private and governmental hospitals obtained accreditation from different international accreditation bodies including the Joint Commission International (JCI), Accreditation Canada, and The Australian Council on Healthcare Standards (ACHS). The first hospital in Saudi Arabia to obtain international accreditation was King Faisal Specialist Hospital and Research Centre in the year 2001.

The objective of this study was to assess a developing country accreditation standards (CBAHI standards) against the ISQua principles in order to identify opportunities for improvement of the CBAHI standards

## METHODS

This was a qualitative analysis and assessment of the Saudi accreditation standards using the published ISQua principles for accreditation standards. ISQua principles were chosen because they are a well-respected international organization accepted by many international accreditation bodies including JACHO, ACHS and the Healthcare Accreditation Quality Unit, UK.

ISQua international principles for healthcare standards were developed as a guide for accreditation bodies to develop accreditation standards. ISQua principles consists of six principles, each of which consists of 4-14 sub-principles. ISQua produces guidance and a sample of standards assessment to assist in the interpretation and application of ISQua principle. The principles and sub-principles are rated on a three-point scale of Met, Partially Met and Not Met (Table 1). As shown in Appendix A (available online at www.saudiannals.net) sub-principle 1.8 was rated as Met because CBAHI standards included all areas covered in the explanatory guidance of this sub-principle, including establishing systems for adverse events, medication errors and patients complaints. Sub-principle 2.3 was rated as Partially Met because CBAHI standards addressed the informed consent and patient involvement in the process of care; however, there were deficiencies in covering end-of-life care and patients’ rights to be treated or not to be treated. Sub-principle 3.6 was rated as Not Met because CBAHI standards did not encourage active participation of patients and the community in the planning for the provision of health care services.

**Table 1:** Terminology of standards assessment

Explanation	Assessment
Standards included all areas covered in the ISQua explanatory guidance of the sub-principle	Met
Standards included some areas covered in the ISQua explanatory guidance; however the standards are deficient in addressing other areas covered in the ISQua explanatory guidance of the sub-principle	Partially Met
Standards did not include any area covered in the ISQua explanatory guidance of the sub-principle	Not Met

## RESULTS

### Assessment of CBAHI standards against ISQua principles

The assessment of accreditation standard of accrediting bodies using ISQua principles is an important process to assure accreditation bodies that their accreditation standards meet international principles and to assure their customers and sponsors about the quality of accreditation services they provide. ISQua established seven principles, including fifty-six sub-principles. The detailed assessment is shown in Appendix A. A summary of the assessment is shown in Table 2.

**Table 2:** Summary of the results of CBAHI standards assessment.

Principles	Met	Partially Met	Not Met	Overall rating
Quality Improvement	1.8	1.1, 1.2, 1.3, 1.4, 1.5, 1.6, 1.7, 1.9		Partially Met
Patient/Service User Focus	2.1, 2.2	2.3, 2.4, 2.5, 2.6, 2.7, 2.8, 2.9		Partially Met
Organizational Planning and Performance	3.1, 3.2, 3.3, 3.4	3.5, 3.7, 3.8, 3.9, 3.10	3.6	Partially Met
Safety	4.3, 4.5, 4.6, 4.7, 4.8, 4.9, 4.10,	4.1, 4.2, 4.4		Partially Met
Standards Development	5.2	5.1, 5.5, 5.6, 5.8, 5.14	5.3, 5.4, 5.7, 5.9, 5.10, 5.11, 5.12, 5.13	Partially Met
Standards Measurement	6.1, 6.3,	6.2, 6.4		Partially Met

### Quality Improvement

There is evidence of focus on quality improvement thoughout the standards. A statement of how the standards will contribute to improvements in the health system of Saudi Arabia would be useful to achieve CBAHI aim to improve the quality of health services in Saudi Arabia.

### Patient/Service User Focus

Several standards focus on patients/service users. There is a need to encourage coordination of care and communication from the institution to the general practitioner, or referring hospitals. Evidence-based clinical pathways and guidelines need to be encouraged. There is a need for more standards to consider access for individuals with disabilities and special needs.

### Organizational Planning and Performance

Standards encourage staff to follow evidence based clinical practice guidelines, protocols, and pathways. There are no standards encouraging active participation of patients, consumers and community leaders as partners, in the development of plans for the institution. There is a need for more explicit standards to set long and short term plans and goals considering environmental and financial factors with the monitoring of the progress in achieving these plans and goals and objectives through defined activities being measured and reported on a regular basis. Coordination with external services should be encouraged.

### Safety

Several standards cover important aspects of safety. There is a need for more explicit standards that emphasize the need to have a risk management plan and to coordinate and plan risk management activities. There is also a need for standards for workload monitoring, stress management and waste handling.

### Standards Development

Standards development was not explicitly described. Membership of the CBAHI board includes different governmental health organizations and a representative of the private sector. Patient input was not incorporated. There is no clear process for revision and feedback of the standards. The structure of the standards is based on the different clinical services rather than having a patient-focused or management-focused structure. They are poorly organized, with no subheadings, and there is significant repetition, complexity and ambiguity. The standards lack notes to explain the intent of each standard, and there is a lack of measurable elements for the different standards.

### Standards Measurement

Evidence indicates that standards have the potential to enable consistent measurement. However, there are opportunities to provide more guidelines to assist users to be more consistent in rating health care organizations. There is no clear process of health care organization to provide feedback about the standards and the measurement of the standards.

## DISCUSSION

The CBAHI standards have a good focus on quality improvement and patients/service users; however, there is a need to link these items to the health system of Saudi Arabia. The standards emphasize the importance of planning, but do not involve patients and community leaders in the planning process. Standards cover important aspects of safety, but there is a need for more explicit standards to coordinate risk management activities. Standards development was not well described. The structure of standards is not well organized and there is significant repetition, lack of sub-headings, a lack of notes describing the intent of standards, and a lack of measurable indicators for majority of standards. Generally standards measurement provides a consistent measurement of the performance of health organizations; however, there is no clear process to provide feedback about the standards. CBAHI standards should be based on evidence and should encourage evidence-based practice.^7^

This study is limited because of the fact that we could not compare our assessment of CBAHI standards with the assessment of the local standards of other countries as there are not published articles reporting on the evaluation of local accreditation standards against ISQua principles. In conclusion, the majority of CBAHI standards did not meet or only partially met the majority of ISQua principles. CBAHI standards development is not well described without a clear process to revise the standards. There is significant repetition and ambiguity. In addition CBAHI standards lack measurable elements for each standard. There is a need to encourage assessments of local accreditation programs in other countries to enable developing countries to assess, compare and improve local accreditation programs.

## References

[1] Shaw CD (2004). Toolkit for Accreditation Programs. The International Society for Quality In Health Care, Australia.

[2] Montagu D (2003). Accreditation and other external quality assessment systems for healthcare: Review of experience and lessons learned. http://www.dfidhealthrc.org/publications/health_service_delivery/Accreditation.pdf.

[3] Shaw CD (2000). External quality mechanisms for health care: summary of the ExPeRT project on visitatie, accreditation, EFQM and ISO assessment in European Union countries. External Peer Review Techniques. European Foundation for Quality Management. International Organization for Standardization. Int J Qual Health Care.

[4] CBAHI web site. Central board for Accreditation of Healthcare Institutions (CBAHI).

[5] ISQUA. International Principles for Healthcare Standards a Framework of Requirements for Standards.

[6] www.isqua.org.

[7] Greenfield D, Braithwaite J (2009). Developing the evidence base for accreditation of healthcare organisations: a call for transparency and innovation. Qual Saf Health Care.

